# Association between erectile dysfunction and *Helicobacter pylori*, folic acid, vitamin B12, and homocysteine: a cross-sectional study

**DOI:** 10.1093/sexmed/qfac018

**Published:** 2023-03-01

**Authors:** Jiangnan Xu, ZhenYu Xu, Huixing Pan, Zhengdong Zhou

**Affiliations:** Department of Urology, The First People’s Hospital of Yancheng, Yancheng 224006, China; Department of Urology, Kunshan Chinese Medicine Hospital Affiliated to Nanjing University of Chinese Medicine, Suzhou 215399, China; Department of Urology, The First People’s Hospital of Yancheng, Yancheng 224006, China; Department of Urology, The First People’s Hospital of Yancheng, Yancheng 224006, China

**Keywords:** erectile dysfunction, *H pylori*, folic acid, homocysteine, vitamin B12

## Abstract

**Background:**

Previous studies have shown that *Helicobacter pylori* (Hp) infection is associated with erectile dysfunction (ED), but the mechanism is unclear.

**Aim:**

To assess the relationship between ED and Hp, folic acid (FA), vitamin B12 (B12), and homocysteine (HCY).

**Methods:**

This study included 84 patients with ED and 42 healthy men. We adopted an IIEF-5 score <21 (5-item International Index of Erectile Function) as the diagnostic criterion for ED, and the RigiScan monitoring device was used to preliminarily screen for and rule out psychogenic ED.

**Outcomes:**

Levels of Hp immunoglobulin G (Hp-IgG) titer, FA, B12, and HCY were compared between the ED group and the non-ED group, and the correlation between the indicators was evaluated.

**Results:**

The median Hp-IgG titer was higher in the ED group than the control group (32.34 vs 20.88, *P* < .001). The ED group had lower median levels of B12 (195 vs 338, *P* < .001) and FA (4.66 vs 10.31, *P* < .001) and a higher median level of HCY (12.7 vs 8.1, *P* < .001). Multivariate logistic regression analysis showed that the level of FA (odds ratio, 0.111; 95% CI, 0.031-0.399; *P* < .001) was an independent risk factor for ED. Specifically, FA level was significantly higher in the moderate ED group than the severe ED group, which had a higher median Hp-IgG titer and lower level of B12; although not significant, this was still a clinical trend. Hp-IgG titer was negatively correlated with levels of FA (*r* = −0.601, *P* < .001) and B12 (*r* = −0.434, *P* < .001) and with the IIEF-5 score (*r* = −0.382, *P* < .001) and positively correlated with HCY (*r* = 0.69, *P* < .001).

**Clinical Implications:**

The ED group had higher levels of Hp-IgG titer and HCY and lower levels of B12 and FA.

**Strengths and Limitations:**

This study is the first to link Hp infection, FA, B12, and HCY and further explain the relationship between these indicators and the underlying pathologic mechanisms that jointly cause ED. The limitation is that our study was based on Hp-IgG titers, which do not necessarily represent the full extent of Hp infection, despite the avoidance of invasive testing.

**Conclusion:**

Hp infection might lead to decreased FA and B12 and then increased HCY, which might be a mechanism leading to ED. Hp eradication or FA and B12 supplementation might have certain clinical value in the treatment of vascular ED.

## Introduction

Erectile dysfunction (ED) refers to the persistent inability to initiate or maintain a rigid erection of the penis for satisfactory sexual intercourse, which can seriously impair the physical and mental health of the sufferers and their partners as well.^1^^,^^2^ ED is a major component of male sexual dysfunction, with a prevalence of 52% in men aged 40 to 70 years, and is expected to account for 322 million cases worldwide by 2025.^3^^,^^4^

Erection is a complex process that requires the coordination of the neuroendocrine system, smooth muscle, and vascular endothelial cells.^5^ Although any abnormality in these parts can lead to ED, in essence, erection is a vascular event, and vascular smooth muscle and endothelial integrity are essential.^6^ Previous studies have shown that homocysteine (HCY) can inhibit endothelial nitric oxide synthase (eNOS) to a certain extent and promote eNOS uncoupling.^7^^,^^8^ Since ED and endothelial dysfunction are associated with reduced expression and activation of eNOS, hyperhomocysteinemia (HHcy) is considered an independent risk factor for ED.^9^^,^^10^ In our previously published study, we found that levels of folic acid (FA) and vitamin B12 (B12), critical cofactors of HCY metabolism, were significantly lower in patients with ED than in the healthy control, which could lead to HCY accumulation and may be the upstream cause of HHcy.^11-13^ However, the reasons for the lower FA and B12 levels in patients with ED and the causal relationship between them remain controversial. Although previous studies noted that it may be related to staying up late and smoking, these reasons do not fully explain the phenomenon.^11^^,^^14^

Interestingly, a recent study showed that *Helicobacter pylori* immunoglobulin G (Hp-IgG) titers were significantly higher in patients with ED than in the healthy control; despite its interpretation from the perspective of inflammation and atherosclerosis, the study still gave us great insight.^15^ Hp is particularly adapted to live in the stomach lumen and is thought to infect about 50% of the world’s population.^16^ Despite the large size of the infected population, those infected often seek medical attention for digestive and anemic symptoms, while other adverse effects go unnoticed.^17^^,^^18^ As early as 2002, Tamura et al pointed out that Hp infection would lead to malabsorption of B12 and FA and increased HCY levels, which would in turn promote atherosclerosis.^19^ Subsequent studies have validated the effects of Hp infection on FA, B12, and HCY but have not linked this phenomenon to ED.^20-22^

Therefore, we hypothesized that Hp infection might lead to lower FA and B12 levels, affect HCY metabolism, and eventually contribute to the development of ED. To verify this conjecture, we detected levels of Hp-IgG titer, FA, B12, and HCY between the ED group and healthy control group, explored the relationship between Hp infection and ED, and tried to explain the internal association between the stated indicators.

## Methods

### Study design

By referring to the sample size calculation method provided by the previous study, our study was cross-sectional and 2-sided (α = 0.05, β = 0.2). We estimated that at least 68 participants with ED and 18 healthy men were required for this study.^23^ Our study recruited 126 participants from our centers between April 2022 and July 2022. Participants with ED were recruited consecutively from the outpatient Department of Andrology and were required to meet the established inclusion and exclusion criteria. We consecutively recruited healthy men who underwent physical examinations in our medical center during the same period as controls. Both groups were sexually active men with partners. This study was approved by the Ethics Committee of our center. All participants signed the informed consent (approval 2022/146).

### Data collection

A detailed examination of all participants’ medical histories was conducted and consisted of the following:

- Any medical history that may be a risk factor for ED, especially hypertension, diabetes, coronary heart disease, liver, kidney, and nervous system diseases
- History of trauma, surgery, or medication that may affect erectile function
- Factors that might cause psychological ED, such as a history of depression and psychiatric disorders
- Any other disease that can cause the relevant serologic abnormalities, such as gastrointestinal diseases, pituitary neoplasms, and thyroid diseases
- A history of ulcerative gastric disease and anemia

All enrollees underwent a comprehensive reproductive system examination to assess testicular and penile development. The erectile function of all participants was assessed with the 5-item International Index of Erectile Function (IIEF-5).^24^ Scores between 22 and 25 were assessed as non-ED, 17 to 21 as mild, 12 to 16 as mild to moderate, 8 to 11 as moderate, and 1 to 7 as severe ED.^25^ We used the audiovisual sexual stimulation and RigiScan test with oral phosphodiesterase 5 inhibitors to assess penile rigidity and tumescence to preliminarily screen for and rule out psychogenic ED. The definition of a typical result is based on the Chinese population criteria proposed by basal stiffness >60% sustained ≥8.75 min, average event stiffness of tip ≥43.5% and base ≥50.5%, and average maximum stiffness of tip ≥62.5% and base ≥67.5%.^26^

Venous blood samples were collected from all participants between 8 am and 10 am after a 12-hour overnight fast to measure fasting blood glucose, total cholesterol, total triglycerides, B12, HCY, FA, and Hp-IgG titer. Serum B12, FA, and HCY levels were determined by chemiluminescence immunoassay (ADVIA Centaur XP; Siemens Medical Diagnostics). Hp-IgG titers were quantified by enzyme-linked immunosorbent assay with commercially available kits (Abnova), following the manufacturer’s guidelines.

### Inclusion and exclusion criteria

For the inclusion criteria, the experimental group consisted of sexually active men aged 20 to 55 years who complained of ED symptoms for >6 months and were diagnosed with ED by IIEF-5 score. Age-matched healthy men constituted the control group. All participants had signed informed consent.

Exclusion criteria were as follows:

- Any medical history that may be a risk factor for ED, especially hypertension, diabetes, and coronary heart disease, as well as liver, kidney, and nervous system diseases
- Trauma and surgical history that might affect erectile function, such as pelvic fracture and penile surgery
- Drug abuse (opioids, cocaine) and use of drugs that might affect erectile function, such as selective serotonin reuptake inhibitors
- Significant dyslipidemia (total cholesterol >200 mg/dL and/or total triglycerides >150 mg/dL) and hormonal abnormalities such as hypogonadism (total testosterone <2 ng/mL) and hyperprolactinemia (serum prolactin >35 ng/mL)
- Psychogenic ED diagnosis by audiovisual sexual stimulation and RigiScan test

### Statistical analysis

We performed statistical assessments using SPSS version 25.0 (IBM). Continuous variables that were not normally distributed were presented as medians (first and third quartiles), and comparisons between groups were performed through the Mann-Whitney *U* test. Continuous variables with normal distribution are expressed as mean ± SD. Coefficients of variation (CVs) were used to demonstrate the degree of variation in the data. Comparison between groups was analyzed by the independent-sample *t* test. Multivariate logistic analysis was used to determine independent risk factors. Correlation data are expressed as Spearman correlation coefficients.

## Results

According to the established criteria, a total of 84 patients with ED were enrolled in the experimental group, including 47 with moderate ED and 37 with severe. The control group included 42 healthy men. The degree of variation of each index was as follows: CV (Hp) = 0.3887, CV (FA) = 0.4626, CV (HCY) = 0.8054, CV (B12) = 0.4508; all were moderate variation. There were no significant differences in age, body mass index, chronic smoking rates, total cholesterol, total triglycerides, and fasting blood glucose between the groups (Table 1). The ED group had a higher median Hp-IgG titer (32.34 vs 20.88 arbitrary units [arbU]/mL, *P* < .001). The ED group also had lower median levels of B12 (195 vs 338 pmol/L, *P* < .001) and FA (4.66 vs 10.31 ng/mL, *P* < .001) and a higher median level of HCY (12.7 vs 8.1 μmol/L, *P* < .001). Univariate and multivariate logistic regression analysis showed that the level of FA (odds ratio, 0.111; 95% CI, 0.031-0.399; *P* < .001) was an independent risk factor for ED (Table 2).

**Table 1:** Comparison of basic indicators between ED group and control group.^a^

	**ED group (n = 84)**	**Control group (n = 42)**	***P* value**
Age, y	31 (28-34)	30 (25-32)	.189
Body mass index, kg/m^2^	24.43 ± 2.45	23.26 ± 1.81	.070
IIEF-5 score	8 (5-10)	23 (22-24)	<.001^*^
Chronic smoker	24 (28.57)	7 (16.67)	.189
Total triglycerides, mmol/L	1.07 (0.75-1.35)	1.01 (0.94-1.12)	.470
Total cholesterol, mmol/L	4.57 (4.09-5.14)	4.38 (4.12-4.92)	.086
Fasting blood sugar, mmol/L	5.60 (5.50-5.80)	5.45 (5.38-5.72)	.125
Vitamin B12, pmol/L	195.00 (165.00-288.00)	338.00 (322.00-315.00)	<.001^*^
Homocysteine, umol/L	12.70 (9.80-23.78)	8.10 (7.12-9.23)	<.001^*^
Folic acid, ng/mL	4.66 (3.59-6.07)	10.31 (9.29-10.91)	<.001^*^
Hp-IgG, arbU/mL	32.34 (21.04-56.03)	20.88 (17.42-26.56)	<.001^*^

Abbreviations: arbU, arbitrary units; ED, erectile dysfunction; Hp-IgG, *Helicobacter pylori*–specific immunoglobulin G; IIEF-5, 5-item International Index of Erectile Function.

a Values are presented as median (first-third quartiles), mean ± SD, or No. (%).

^*^ *P* < .05.

**Table 2:** Univariate and multivariate logistic regression analysis with erectile dysfunction as the outcome measure.

	**Univariate**			**Multivariate**		
	***B***	**OR (95% CI)**	***P* value**	***B***	**OR (95% CI)**	***P* value**
Hp-IgG	0.140	1.150 (1.096-1.206)	**<.001** ^*^	−0.051	0.950 (0.837-1.078)	.426
Folic acid	−2.102	0.122 (0.440-0.343)	**<.001** ^*^	−2.196	0.111 (0.031-0.399)	**<.001** ^*^
Homocysteine	0.742	2.101 (1.536-2.873)	**<.001** ^*^	0.303	1.354 (0.707-2.594)	.361
Vitamin B12	−0.008	0.992 (0.988-0.996)	**<.001** ^*^	0.001	1.005 (0.992-1.009)	.921

Abbreviations: Hp-IgG, *Helicobacter pylori*–specific immunoglobulin G; OR, odds ratio.

^*^ *P* < .05.

**Figure 1:**
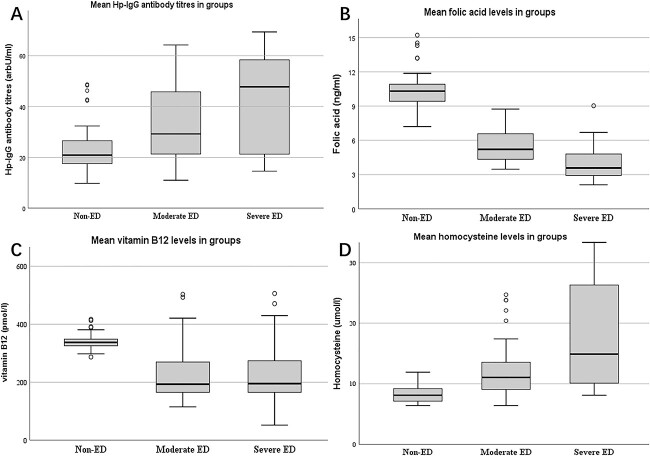
Folic acid, vitamin B12, homocysteine levels, and Hp-IgG antibody titers in the non-ED, moderate ED, and severe ED groups. ED, erectile dysfunction; Hp-IgG, *Helicobacter pylori* immunoglobulin G.

The FA level in the moderate ED group was higher than that in the severe ED group (5.20 vs 3.58 ng/mL, *P* < .001). There were no significant differences in median levels of B12 (201 vs 195 pmol/L, *P* = .682), HCY (11.7 vs 14.9 μmol/L, *P* = .077), and Hp-IgG titer (29.23 vs 47.76 arbU/mL, *P* = .138) between the moderate and severe ED groups (Table 3). Although the differences were not statistically significant, we can see certain clinical trends in Figure 1. With the increase of ED severity, the levels of FA and B12 decreased, and the levels of Hp-IgG and HCY increased.

**Table 3:** Comparison of clinical and biochemical indexes between moderate and severe ED.^a^

	**Moderate ED group (n = 47)**	**Severe ED group (n = 37)**	***P* value**
IIEF-5 score	10 (9-11)	5 (4-7)	<.001^*^
Chronic smoker	24 (28.57)	7 (16.67)	.189
Vitamin B12, pmol/L	201.00 (165.00-304.00)	195.00 (164.00-277.00)	.682
Homocysteine, umol/L	11.70 (9.30-20.40)	14.90 (10.10-28.40)	.077
Folic acid, ng/mL	5.20 (4.31-6.69)	3.58 (2.91-4.93)	<.001^*^
Hp-IgG, arbU/mL	29.23 (20.98-46.89)	47.76 (20.55-58.53)	.138

Abbreviations: arbU, arbitrary units; ED, erectile dysfunction; Hp-IgG, *Helicobacter pylori*–specific immunoglobulin G; IIEF-5, 5-item International Index of Erectile Function.

a Values are presented as median (first-third quartiles) or No. (%).

^*^ *P* < .05.

Correlation analysis showed that Hp-IgG titer was negatively correlated with levels of FA (*r* = −0.601, *P* < .001) and B12 (*r* = −0.434, *P* < .001) and with IIEF-5 score (*r* = −0.382, *P* < .001) and positively correlated with HCY (*r* = 0.69, *P* < .001). IIEF-5 score was positively correlated with levels of FA (*r* = 0.842, *P* < .001) and B12 (*r* = 0.362, *P* < .001) and negatively correlated with level of HCY (*r* = −0.398, *P* < .001) (Table 4).

**Table 4:** Correlation analysis.

	**Hp-IgG**		**IIEF-5 score**	
	**Correlation, *r***	***P* value**	**Correlation, *r***	***P* value**
IIEF-5 score	−0.382	<.001^*^	—	—
Folic acid	−0.601	<.001^*^	0.842	<.001^*^
Vitamin B12	−0.434	<.001^*^	0.361	<.001^*^
Homocysteine	0.690	<.001^*^	−0.398	<.001^*^

Abbreviations: Hp-IgG, *Helicobacter pylori*–specific immunoglobulin G; IIEF-5, 5-item International Index of Erectile Function.

^*^ *P* < .05.

## Discussion

**Figure 2:**
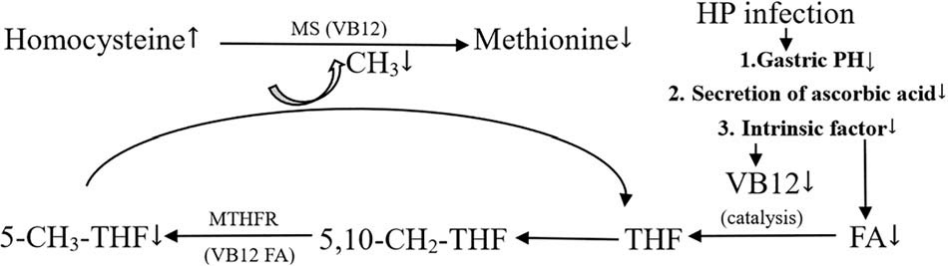
Metabolic pathways of folic acid, vitamin B12, and homocysteine and their relationship with *Helicobacter pylori* infection. CH3, methyl; FA, folic acid; Hp, *Helicobacter pylori*; MS, methionine synthase; MTHFR, methylene tetrahydrofolate reductase; THF, tetrahydrofolate; VB12, vitamin B12.

Hp is a spiral-shaped gram-negative bacterium that infects more than half the world’s population; the most common routes of Hp transmission are oral to oral and fecal to oral.^27^ Although previous studies mainly focused on upper gastrointestinal system diseases caused by Hp, extraintestinal diseases caused by Hp began to attract scientific attention until the end of the 20th century. Yağmur et al first found that patients with ED had high levels of Hp-IgG titer.^15^ Our previous studies showed that men with ED have lower FA and B12 levels and a higher HCY level.^11^ This study is the first to link Hp infection, FA, B12, and HCY and further explain the relationship between these indicators and the underlying pathologic mechanisms that jointly cause ED. Given the results of this study, we hypothesized that the absorption disorder of FA and B12 caused by Hp infection contributed to the metabolic disorder of HCY, which caused HHcy and then damaged vascular endothelial function, thus leading to the occurrence of ED (Figure 2).

Several studies have shown that Hp infection affects the absorption of various trace elements, especially B12 and FA, by disrupting gastric secretion and attenuating acidification functions.^20^^,^^22^^,^^28^ Our study also revealed that patients with ED had higher Hp-IgG titer levels and lower FA and B12 levels, which were significantly negatively correlated with Hp-IgG titer. The specific mechanism of the impairment of FA and B12 absorption caused by Hp infection is still unclear, and there are several possibilities, as shown in Figure 2. First, the Hp-induced decrease in gastric acid secretion may not only lead to difficulties with the critical splitting of B12 and FA from food binders but also interfere with the transfer of food cobalamin to R-binders.^29^ Second, Hp infection can lead to a decrease in intrinsic factor secretion.^22^ Finally, a decrease in ascorbic acid secretion by the gastric mucosa and an increase in gastric pH affect B12 and FA absorption.^21^ Thus, patients with Hp infection may be in a state of low FA and B12 levels due to absorption disorders.

Mendall et al first reported a positive association between Hp infection and coronary heart disease, and subsequent large meta-analyses confirmed this association.^30^ Coronary artery disease and vasogenic ED are different manifestations with similar pathologic mechanisms. Some studies have pointed out that because penile artery atherosclerosis occurs even earlier than coronary artery atherosclerosis, vascular ED can be used as an early indicator of cardiovascular disease.^31-33^ Yağmur et al pointed out that Hp infection may be involved in vascular endothelial injury and atherosclerosis of the penile artery, but the specific mechanism is still unclear.^15^

Our study revealed that one of the mechanisms of penile vascular endothelial injury may be related to HCY metabolism disorder caused by low levels of FA and B12 caused by Hp infection. Previous studies have shown that eNOS activation and expression are inhibited and eNOS decoupling is promoted in patients with HHcy, which can cause endothelial dysfunction and lead to ED.^34^ As shown in Figure 2, B12 and FA are important cofactors in the remethylation of HCY to methionine by methylene tetrahydrofolate reductase. FA and B12 deficiency could hinder HCY metabolism, leading to HCY accumulation and causing HHcy, which is consistent with our findings. In addition, HCY methylated to methionine can be further converted to S-adenosyl methionine, which is an important methyl donor in the process of DNA methylation.^35^ Therefore, HCY metabolism disorder caused by low FA and B12 levels caused by Hp infection may decrease DNA methylation, hinder DNA repair, and induce endothelial cell apoptosis, thereby contributing to the development of ED to some extent.

FA is involved in not only the metabolism of HCY but also the raw material of tetrahydrofolate (THF), and B12 catalyzes the formation of THF.^36-38^ Specifically, B12 catalyzes sufficient FA to generate sufficient THF, which is essential for maintaining tetrahydrobiopterin (BH4) levels. When BH4 is abundant, the affinity of NADPH oxidase (nicotinamide adenine dinucleotide phosphate) for eNOS is enhanced, and the oxidation of L-arginine to L-citrulline is accelerated to provide electrons for nitric oxide (NO) generation.^39^ NO is a relaxation factor synthesized by eNOS, which plays an essential role in the activation and maintenance of an erection.^9^ When FA and B12 deficiency leads to BH4 deficiency, NO production decreases, which in turn leads to ED. Therefore, FA deficiency plays an important role in the development of ED through multiple pathways. Our study also showed that the decreased level of FA was an independent risk factor for ED. Hp-IgG might be an independent risk factor for ED in univariate logistic regression, but it lost statistical significance after multivariate regression, which may be due to the insufficient sample size and needs further exploration.

The results of this study may provide some references for the treatment of ED, including eradication of Hp infection and supplementation of FA and B12. There are still some controversies about the cutoff value of Hp-IgG titers for Hp infection in previous studies, but it is generally distributed between 35 and 40 arbU/mL.^15^^,^^40^^,^^41^ Hp is often treated with combination therapy, including proton pump inhibitors, clarithromycin, amoxicillin, and metronidazole. Serin et al performed Hp eradication therapy in 65 patients with Hp infection, and the eradication treatment was successful in 33 patients. The results showed that the levels of B12 and FA in 65 patients were increased in varying degrees after 2 to 3 months of treatment and that the increase was statistically significant in patients with complete eradication.^42^ As early as 2000, Kaptan et al found that >40% of patients with B12 deficiency anemia could significantly increase their B12 levels to the reference level after radical treatment of Hp.^43^ Agostini et al reported that 400 μg/d of FA combined with inositol improved erectile function in diabetic patients.^44^ Elshahid et al subsequently reported an improvement in median IIEF-5 score from 6 to 14 in 50 patients with ED after continuous supplementation with 500 μg of FA for 3 months; serum HCY level also significantly decreased.^45^ Given our results and the cited studies, we conclude that eradication of Hp and supplementation of FA and B12 might be safe and effective strategies for treating ED. However, due to the lack of relevant studies, large-scale randomized controlled trials are still needed for further verification.

Admittedly, there are still flaws in our research. First, the assessment of ED severity is based on IIEF-5 score, which might introduce bias. Second, our study was based on Hp-IgG titers, which do not necessarily represent the full extent of Hp infection, despite the avoidance of invasive testing. Third, we did not analyze the participants’ hemoglobin levels. Although the relative deficiency of FA and B12 is mostly within the physiologic reference range, there is still a risk of anemia. Fourth, In such a young cohort, some patients with psychogenic ED or comorbid psychological factors were not excluded, which may have biased the results somewhat. Fifth, our results are statistically significant in the case of moderate variation; whether the correlation is reduced in the case of high variation needs to be verified with a larger sample. Finally, our study is cross-sectional and can only find associations and make appropriate etiologic guesses based on known pathophysiologic mechanisms.

In conclusion, Hp infection might lead to decreased FA and B12 levels, which in turn affect HCY metabolism, cause HHcy, and then damage vascular endothelial function, leading to ED. Hp eradication or FA and B12 supplementation might have certain clinical value in the treatment of vascular ED.

## Funding

None declared.

*Conflicts of interest:* The authors declare that they have no conflict of interest.

## Data availability

Raw data can be requested from the corresponding author if there is a reasonable request.
